# Supplementary material for: A whole-animal phenotypic drug screen identifies suppressors of atherogenic lipoproteins
Source: eLife. 2026 Aug 27;14:RP105314. doi: 10.7554/eLife.105314 (PMC13521566; doi:10.7554/eLife.105314)
Supplement: Supplementary file 2 [file elife-105314-code1.zip › JHDL_primary_result/Sulconazole/Sulconazole_JHDL_14_020_030_2019_08_23_11_22.pdf]

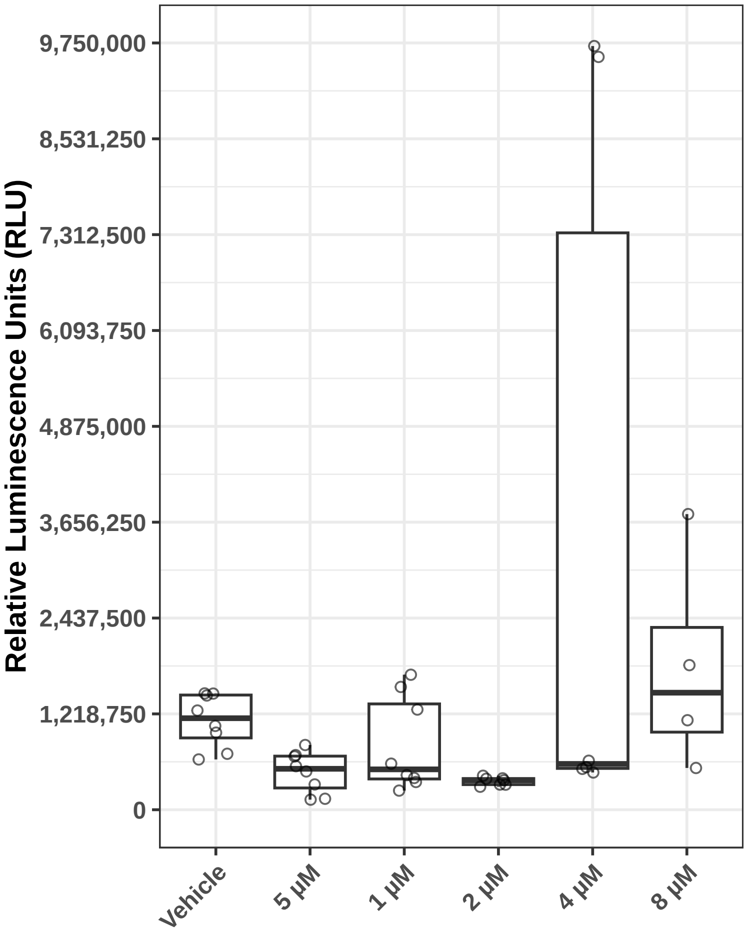

Relative Luminescence Units (RLU)
9,750,000
8,531,250
7,312,500
6,093,750
4,875,000
3,656,250
2,437,500
1,218,750
0
Vehicle
5 µM
1 µM
2 µM
4 µM
8 µM
